# ﻿A detailed comparison of two species in the genus *Potamanthus* Pictet, 1843 from China (Ephemeroptera, Potamanthidae)

**DOI:** 10.3897/zookeys.1125.89219

**Published:** 2022-10-26

**Authors:** Wen-Juan Li, Chang-Fa Zhou

**Affiliations:** 1 The Key Laboratory of Jiangsu Biodiversity and Biotechnology, College of Life Sciences, Nanjing Normal University, Nanjing 210023, China Nanjing Normal University Nanjing China

**Keywords:** China, *Potamanthus*, *P.huoshanensis*, *P.luteus*, morphology

## Abstract

Photographs and details of structures of two *Potamanthus* species, *P.huoshanensis* Wu, 1987 and *P.luteus* (Linnaeus, 1767), are presented for the first time. Here, based upon Chinese specimens of those species, all external structures are illustrated digitally and compared. The results and photos clearly show that the adults of the two species are different in wing color and genitalia shape, and that their nymphs have different mandibular tusks and forelegs. Specifically, *P.luteus* has a more colorful body and wings, longer penes and nymphal mandibular tusks but shorter foretarsi than those of *P.huoshanensis*. This comparison not only confirms the differences between these two similar species, but also supports the updated generic delineations of *Potamanthus* and *Potamanthodes*.

## ﻿Introduction

The Palearctic genus *Potamanthus* Pictet, 1843 comprises only two species and one subspecies (Bae and McCafferty 1991; Kluge 2004; Li and Zhou 2022). The first one, *P.luteus* (Linnaeus, 1767), is widely distributed from northern Africa and Europe to northeastern Asia, and its morphology has been described and mentioned by a long series of researchers (see Bauernfeind and Soldán 2012 and references therein). However, only Bae and McCafferty (1991) provided photos of this species, but no comprehensive, detailed photographs had so far been presented to show its exact characters. Further, *P.luteus* was divided into two subspecies by Bae and McCafferty (1991) using few structures, such as the shape of the anterolateral corners of the nymphal pronotum, the vestigial apical spines on the forefemora and forking point of the medius anterior (MA) in the imaginal forewings.

In contrast to *P.luteus*, the second species in the genus, *P.huoshanensis* Wu, 1987, has a very narrow distribution. Up until now, it has been found only at one site in China and two sites in Japan (Wu 1987a, 1987b; Ishiwata 2001). Only the wings and a drawing of the nymphal habitus of this species had been provided so far, by Bae and McCafferty (1991). In addition, Wu (1987a) and Bae and McCafferty (1991) regarded this species as very similar to *P.luteus*, the latter authors even identifying Japanese *Potamanthus* materials as belonging to *P.luteus*. Thus, proper photographic documentation of *P.huoshanensis* would not only reveal the real characters of this species but also show differences with the similar *P.luteus*.

The generic circumscription and phylogeny of the genus *Potamanthus* has been changing. Bae and McCafferty (1991) downgraded the taxon *Potamanthodes* to a subgenus of *Potamanthus*. Differently, Kluge (2004) placed it as a member of another genus, *Rhoenanthus* Eaton, 1881. Recently, Li and Zhou (2022) reinstated the taxon *Potamanthodes* as an independent genus. With details of the two species in the genus *Potamanthus* and other recent related reports (Han et al. 2021; Kwanboon et al. 2021), differences among these three taxa will be clarified.

Here, we compare Chinese specimens of *P.luteus* to the types of *P.huoshanensis*, provide photographs of imaginal and nymphal structures of both species, document fine characters useful to differentiate these two species in the genus *Potamanthus*. The results support our proposal to reinstate this genus in a previous work (Li and Zhou 2022).

## ﻿Material and methods

### ﻿Material examined

***Potamanthushuoshanensis* Wu, 1987**

1♂ imago (**Holotype**), 10 nymphs, 4 ♂♂ imagoes, 20 ♀♀ imagoes (**Paratypes**), Zhufoan Town, Huoshan county, Anhui Province, China, 31°24'59"N, 116°10'30.40"E, 1983-VI-11–13, collected by Xing-Yong WU; other materials: 2 nymphs, 15 ♀♀ imagoes, 1984-VI-11, other information as for the types.

***Potamanthusluteus* (Linnaeus, 1767)**

7 nymphs, 12 ♀♀ imagoes, Nancha county, Heilongjiang Province, China, 47°7'48"N, 129°16'48"E, 1984-VII-26–29, collected by Xing-Yong WU; 1 nymph, Mohe county, Heilongjiang Province, China, 52°58'12"N, 122°31'48"E, 2007-VIII-14, collected by Shi-Lei WANG, Hui XIE; 100 ♂♂ imagoes, 200 ♀♀ imagoes, 100 ♂♂ subimagoes, 100 ♀♀ subimagoes, Yanshou county, Heilongjiang Province, China, 45°27'0"N, 128°19'48"E, 2008-VII-14–15, collected by Shi-Lei WANG, Guo ZHAO; 2 nymphs, 1 ♀ imago, Songhua River, Fusong county, Jilin Province, China, 44°41'31"N, 125°57'8.82"E, 2008-VII-26, collected by Shi-Lei WANG, Guo ZHAO.

### ﻿Methods

The nymphs of two species studied in the present paper were collected by hand net, whereas most adults were collected by light trap (using LED and mercury lamps). Some adults were reared from nymphs in the field. The materials are stored in ethanol (about 85%).

All specimens were examined under a stereomicroscope (MshOt MZ81) and photographed with a digital camera coupled to the microscope (Nikon Eclipse 50i). Some small structures, such as gills, mouthparts, terga and legs, were observed and photographed with a microscope camera on temporary slides. All specimens used in this study are deposited in the mayfly collection of the College of Life Sciences, Nanjing Normal University, China.

## ﻿Results

### ﻿ Potamanthus huoshanensis

Taxon classificationAnimaliaEphemeropteraPotamanthidae

Wu, 1987

70800F60-A37D-58A5-B55A-20F3E9996412

Potamanthus (Patamanthus) huoshanensis Wu, 1987b: 421. figs 1–5. Types: nymph, male and female, from Anhui, China.
Potamanthus (Patamanthus) huoshanensis : Bae and McCafferty 1991: 49. figs 15, 95, 113, 126, 139 (nymph, male and female); Ishiwata 2001: 58; Zhou 2013: 202; Zhou et al. 2015: 252.
Potamanthus huoshanensis : Wu et al. 1991: 111. fig. 2 (egg); You and Gui 1995: 116. fig. 123 (male and female).

#### Distribution.

China (Anhui Province); Japan (Yokkaichi city, Lake Biwa).

#### Description.

see Wu (1987b) and Bae and McCafferty (1991).

#### Diagnosis.

This species resembles *Potamanthusluteus* in the main characters of both the adults and the nymphs, which can be differentiated only by very fine structures (Table 1). In the nymph, the labrum of *P.huoshanensis* is slightly narrower than that of *P.luteus* (Fig. 3A, B); the mandibular tusks are indistinctly shorter than in *P.luteus*, and this can be seen in nymphal dorsal views (Figs 2A, B, E, F, 3E–H); the maxillary palpi of both species are similar but different in their length ratio: the ratio in the former species is 1.0: 0.6: 1.0, whereas that of the latter species is 1.0: 0.7: 1.3 (Fig. 3I–L). The two species have a very similar hypopharynx and labia (Fig. 3C, D, M, N). Although the color pattern of examined *P.huoshanensis* has fainted and is pale, the leg lengths are different in the two species: ratio of forefemora: tibiae: tarsi = 1.0: 0.7: 0.6 in *P.huoshanensis* and 1.0: 0.8: 0.6 in *P.luteus*, the former having slightly shorter forelegs and tibiae (Fig. 2I, L). But the midlegs, hindlegs and their claws are very similar (Fig. 2D, H, J, K, M, N).

**Figure 1.**
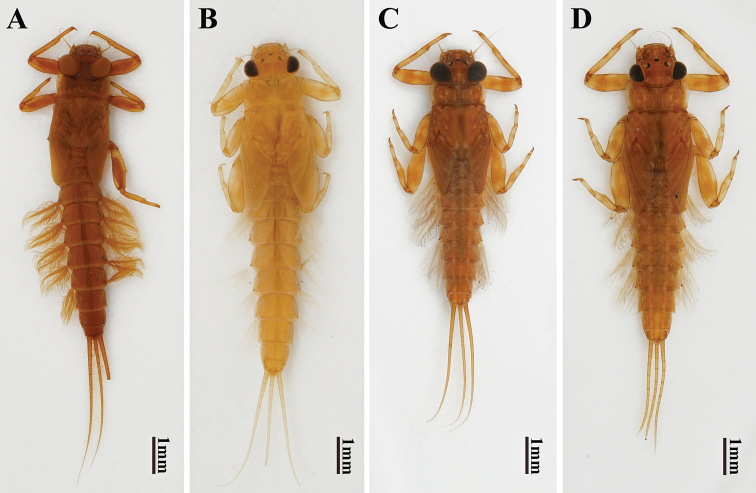
Male and female nymph habitus of two *Potamanthus* species: **A, B***P.huoshanensis* and **C, D***P.luteus*.

**Figure 2.**
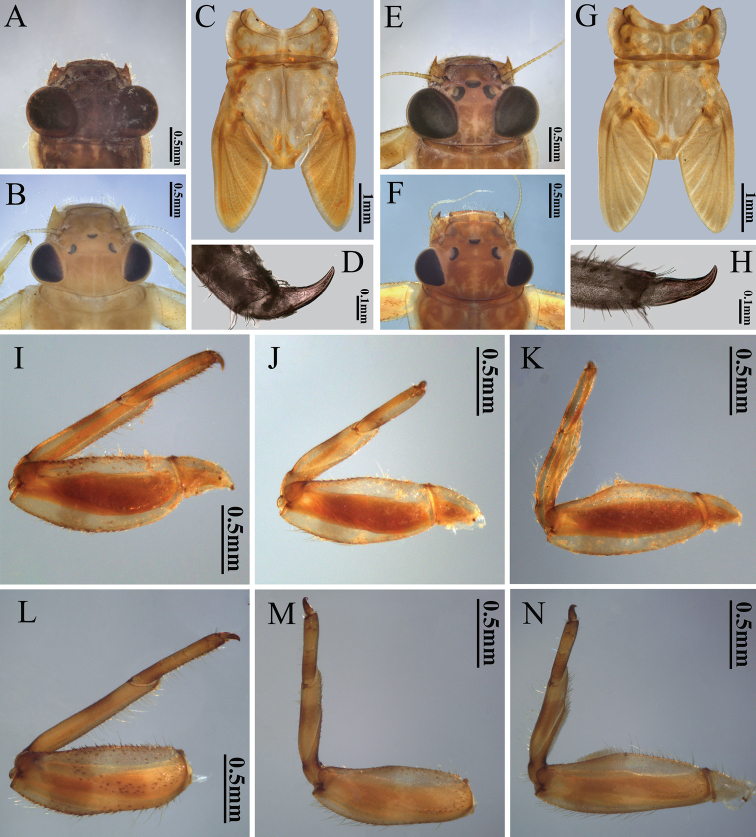
Male and female head, thorax, foreleg claw, foreleg, midleg and hindleg of nymph of two *Potamanthus* species: **A–D, I–K***P.huoshanensis* and **E–H, L–N***P.luteus*.

**Figure 3.**
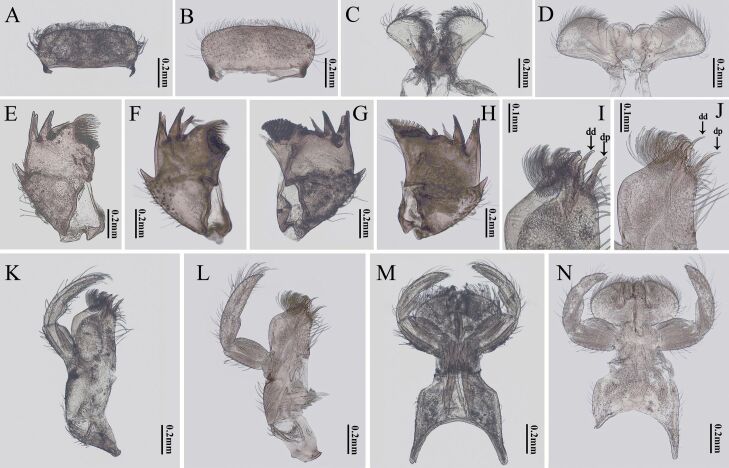
Labrum, hypopharynx, left mandible, right mandible, maxilla and labium of nymph mouthparts of two *Potamanthus* species: **A, C, E, G, I, K, M***P.huoshanensis* and **B, D, F, H, J, L, N***P.luteus*. dd: distal dentisetae; dp: proximal dentisetae.

**Table 1.** Comparison of characteristics of the two *Potamanthus* species.

Characters Species		*P.huoshanensis*	*P.luteus*
Nymph	mandibular tusks	not protruding the labrum	protruding the labrum
	ratio of maxillary palpi from base to apex	1.0: 0.6: 1.0	1.0: 0.7: 1.3
	ratio of forefemora: tibiae: tarsi	1.0: 0.7: 0.6	1.0: 0.8: 0.6
	Pairs of lateral dots on abdominal terga	without	with
Male imago	pigments of crossveins in forewings	vague	clear
	MA: MA_1_	1.0: 0.7	1.0: 0.9
	costal projection of hindwings	blunt	sharp
	distance between two compound eyes	no or very short	half of median ocellus
	Pairs of lateral dots on abdominal terga	without	with
	Penial lobes covered by subgenital plate	partially	no
	Posterior emargination of subgenital plate	shallow	V-shaped cleft
	ratio of forefemora: tibiae: tarsi	1.0: 1.3: 1.6	1.0: 1.6: 1.5
Female imago	pigments of crossveins in forewings	vague	clear
	MA: MA_1_	1.0: 1.0	1.0: 0.9
	Pairs of lateral dots on abdominal terga	without	with

**Figure 4.**
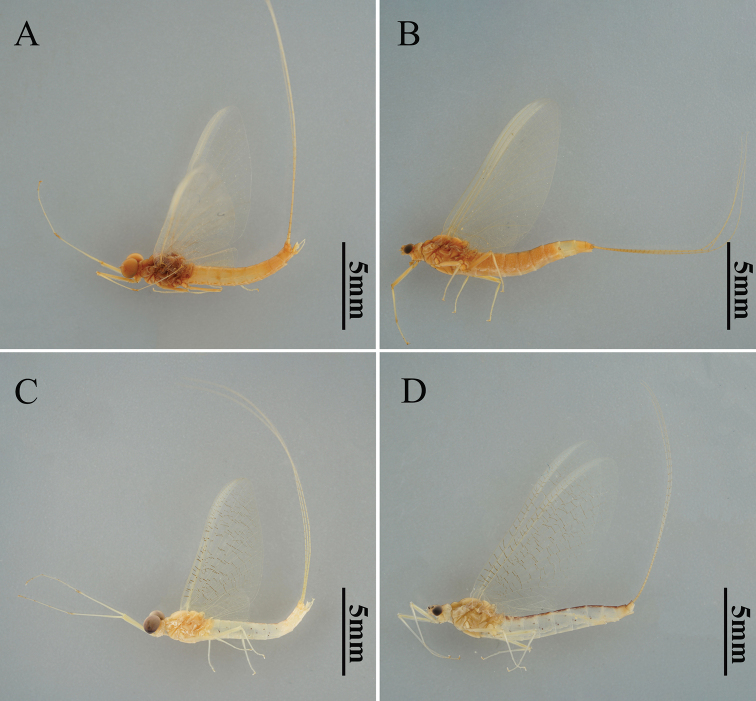
Male and female adult morphology of two *Potamanthus* species: **A, B***P.huoshanensis* and **C, D***P.luteus*.

**Figure 5.**
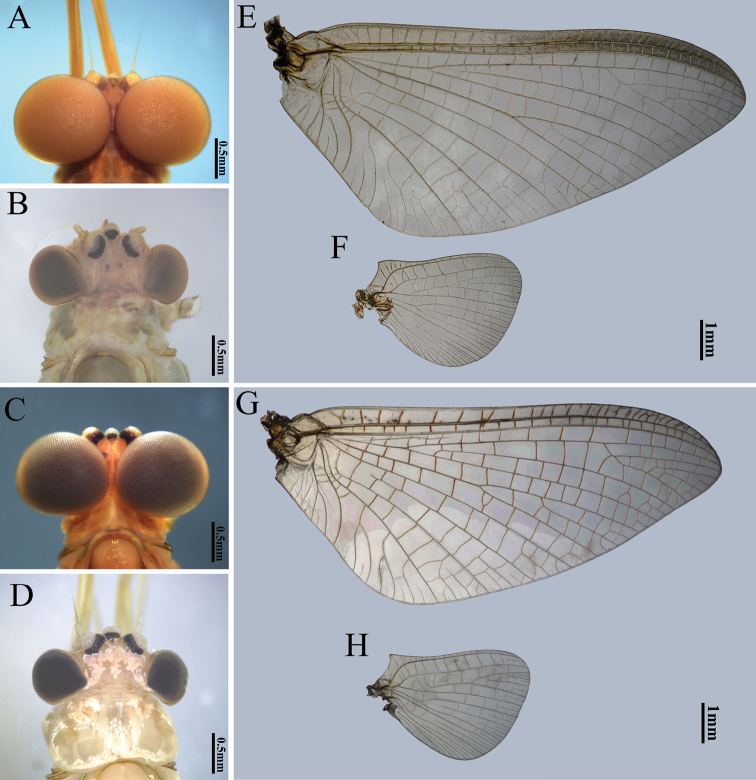
Male head, female head, forewing and hindwing of two *Potamanthus* species: **A, B, E, F***P.huoshanensis* and **C, D, G, H***P.luteus*.

**Figure 6.**
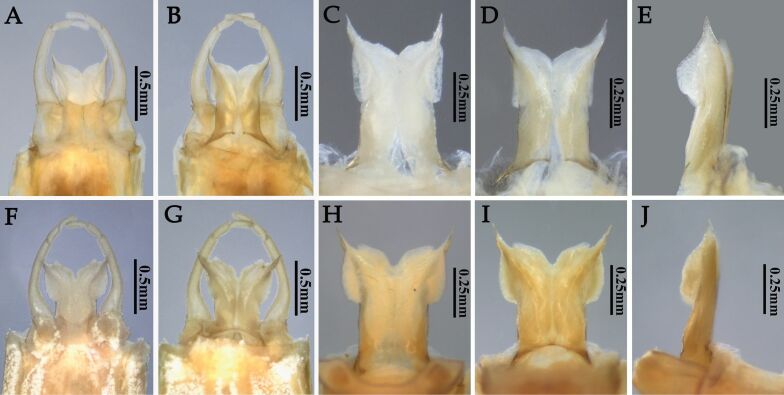
Genitalia (ventral and dorsal view) and penis (ventral, dorsal and lateral view) of two *Potamanthus* species: **A–E***P.huoshanensis* and **F–J***P.luteus*.

**Figure 7.**
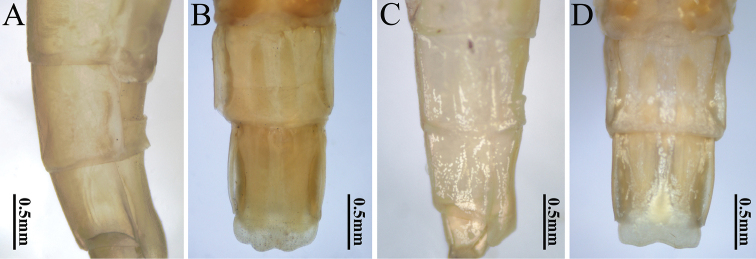
Abdominal segments VII–X (lateral and ventral view) of two *Potamanthus* species: **A, B***P.huoshanensis* and **C, D***P.luteus*.

Males of the two species can be easily separated: (1) the pigments of the crossveins of the forewings of *P.huoshanensis* are almost invisible, but they are clear on the forewings of *P.luteus* (Figs 4A, C, 5E, G); (2) the costal projection of the hindwings are slightly blunter in *P.huoshanensis* than in *P.luteus* (Fig. 5F, H); (3) the compound eyes of *P.huoshanensis* are almost contiguous but they are clearly separated in *P.luteus* (Fig. 5A, C); (4) both the lateral and inner extended lobes of the penis of *P.huoshanensis* are slightly smaller than those of *P.luteus* (Fig. 6C–E, H–J); (5) the penes of *P.huoshanensis* are slightly shorter than those of *P.luteus*: the subgenital plate of *P.huoshanensis* almost covers the base of the penial lobes but the penes of *P.luteus* are longer, with the whole penes completely visible in ventral view (Fig. 6A, B, F, G); (6) the subgenital plate of *P.huoshanensis* has a shallow median emargination, whereas that of *P.luteus* has a clear V-shaped cleft (Fig. 6A–D, F–I); (7) the forking point of the MA in the *P.huoshanensis* forewings is more distal than that of *P.luteus*, with the ratio of MA: MA_1_ = 1.0: 0.7 in the former species and 1.0: 0.9 in the latter (Fig. 5E, G); (8) the foretibiae of *P.huoshanensis* are shorter than in *P.luteus*, with the ratio forefemora: tibiae: tarsi = 1.0: 1.3: 1.6 in *P.huoshanensis* and 1.0: 1.6: 1.5 in *P.luteus* (Fig. 4A, C).

The females of the two species can differentiated by their wing color and the shape of the hindwings, like in the males (Fig. 4B, D). The compound eyes of female *P.luteus* are slightly smaller than those of *P.huoshanensis* (Fig. 5B, D), but the subgenital plates are very similar (Fig. 7).

Although the color of the *P.huoshanensis* material is not clear, the original description of Wu (1987b) and our specimens clearly show that the males, females and nymphs of this species do not have dots on their abdominal terga. In contrast, all stages of *P.luteus* have a pair of dark dots on the abdominal terga (Fig. 4C–D). In addition, *P.luteus* has a longitudinal median reddish band on the abdomen (Fig. 4C–D).

The differences between the two species are listed in Table 1.

### ﻿ Potamanthus luteus

Taxon classificationAnimaliaEphemeropteraPotamanthidae

(Linnaeus, 1767)

585EAD22-73DC-50FA-8759-BC480D1F3739

Ephemera luteus Linnaeus, 1767: 906. Type: England.
Ephemera reticulata Fourcroy, 1785: 351. Synonymized by Eaton (1871: 76).
Baetis mellea Curtis, 1834: 121. Types: subimago. Synonymized by Eaton (1871: 76).
Baetis marginalis Burmeister, 1839: 801. Types: male and female. Synonymized by Eaton (1871: 76).
Ephemera flavicans Rambur, 1842: 296. Types: male and female, from Paris, France. Synonymized by Eaton (1871: 76).
Ephemera chlorotica Rambur, 1842: 296. Types: male and female subimagoes, from Paris, France. Synonymized by Walker (1853: 539).
Potamanthus luteus (Linnaeus): Pictet 1843: 205; Eaton 1884: 79.
Potamanthus ferreri Pictet, 1843: 203. Types: male, from Italy. Synonymized by Bae and McCafferty (1991: 51).
Eucharidis reaumurii Joly & Joly, 1876: 314. Types: nymph. Synonymized by Eaton (1884: 79).
Potamanthus na Imanishi, 1940: 180, fig. 2 (nymph). Synonymized by Bae and McCafferty (1991: 54).
Potamanthus naa Imanishi, 1940: 181 (nymph). Synonymized by Bae and McCafferty (1991: 54).
Potamanthus luteus : Wu 1987a: 336 (female, first record from China); You and Gui 1995: 115, fig. 122 (male); Bauernfeind and Soldán 2012: 634 (adult, nymph, egg).
Potamanthus (Patamanthus) luteus oriens : Bae and McCafferty 1991: 54, fig. 4, 125 (subspecies established); Bae 1997: 408; Zhou 2013: 202; Zhou et al. 2015: 252.
Potamanthus luteus oriens : Quan et al. 2002: 257.

#### Distribution.

China (Heilongjiang and Jilin Province); Palearctic and Oriental. From England east through Europe and Asia Minor, south to North Africa.

#### Description.

see Bae and McCafferty (1991) or Bauernfeind and Soldán (2012).

#### Diagnosis.

see diagnosis of *P.huoshanensis*. Males of this species can be identified by the more distinct color of the wings and penial lobes (Figs 5E–H, 6) and the foretibiae longer than the tarsi (Fig. 4A, C). The nymphs can be distinguished by the slightly larger mandibular tusks, longer foretibiae (Figs 2I, L, 3E–H) and apical segment of the maxillary palpi (Fig. 3K, L).

#### Remarks.

Bae and McCafferty (1991) mentioned that the nymphs of the subspecies *Potamanthusluteusoriens* have very pointed anterolateral projections of the pronotal and vestigial spine-row on the forefemora. In our material, the former character is distinct, and the transverse spine-row was not recognizable, which is consistent with the description of European *P.luteus* provided by Bauernfeind and Soldán (2012). However, we do not know whether this variation is just at the population level or representative of different subspecies or geographical populations, because we have no material from abroad for comparison.

In the present comparison and photos, we can see clearly that *P.huoshanensis* and *P.luteusoriens* are extremely similar in both nymphal and imaginal structures. The differences between them are very slight . Therefore, it is not surprising that Bae and McCafferty (1991) recognized Japanese materials of *P.huoshanensis* as *P.luteusoriens*, which was later corrected by Ishiwata (2001).

The distribution of *P.luteus* is wide, from Africa to Japan. In contrast, *P.huoshanensis* was reported from three allopatric sites in Japan and China. Biogeographic and genetic studies at the population level are required for these species.

At the generic level, the definitions of the genera *Potamanthus* and *Potamanthodes* were updated by Li and Zhou (2022) and confirmed by the characters presented in this study.

### Key to the two *Potamanthus* species

**Nymph**

**Table:** 

1	Mandibular tusks short (not protruding beyond labrum in dorsal view) (Fig. 2A, B, 3E, G); apical segment of maxillary palpi subequal to or shorter than basal one (Fig. 3K)	***P.huoshanensis***
–	Mandibular tusks protruding beyond anterior margin of labrum in dorsal view or subequal (Fig. 2E, F, 3F, H); apical segment of maxillary palpi longer than basal one (Fig. 3L)	***P.luteus***

**Male imago**

**Table:** 

1	Compound eyes almost contiguous (Fig. 5A); foretibiae shorter than tarsi (Fig. 4A); penial lobes partially covered by subgenital plate in ventral view (Fig. 6A); crossveins of forewings without distinct pigments (Figs 4A, 5E)	***P.huoshanensis***
–	Compound eyes separated by half the width of the ocelli (Fig. 5C); foretibiae longer than tarsi (Fig. 4C); penial lobes not covered by subgenital plate, totally visible in ventral view (Fig. 6F); crossveins of forewings with reddish-brown color (Figs 4C, 5G)	***P.luteus***

**Female imago**

**Table:** 

1	Crossveins of forewings without distinct pigments; no distinct dots or markings on abdomen (Fig. 4B)	***P.huoshanensis***
–	Crossveins of forewings with reddish-brown color; mediolongitudinal band and lateral dark dots present on abdomen (Fig. 4D)	***P.luteus***

## Supplementary Material

XML Treatment for Potamanthus huoshanensis

XML Treatment for Potamanthus luteus
